# From design to analysis: A roadmap for predicting distributions of rare species

**DOI:** 10.1111/gcb.16162

**Published:** 2022-03-23

**Authors:** Nigel G. Yoccoz

**Affiliations:** ^1^ Department of Arctic and Marine Biology UiT The Arctic University of Norway Tromsø Norway

## Abstract

Rare species are challenging to study, in part because rarity can take many forms. Jeliazkov et al. guide us through the multiple decisions to be made—from sampling designs to field methods and analytical, integrated models. Improved monitoring methods are needed to improve our understanding of rare species importance for ecosystem structure and functions.

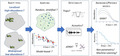

This is a commentary on Jeliazkov et al., 2022, https://doi.org/10.1111/gcb.16114

Who can explain why one species ranges widely and is very numerous, and why another allied species has a narrow range and is rare? (Darwin, 1859)

Naturalists have known for centuries that a majority of species are rare while a few are common. Such a pattern and its underlying causes have long attracted the interest of ecologists (MacArthur, 1957) and statisticians (Fisher et al., 1943). However, like the unhappy families of Tolstoy, every rare species is rare in its own way and we owe Deborah Rabinowitz (1981) a careful analysis of patterns of rarity: she used seven categories, combining distribution, abundance and patchiness. Distinguishing among these categories of rarity is important to analyze and understand the drivers of the distribution and abundance of rare species, their importance for communities and ecosystems, and for the design of efficient conservation policies (Gaston, 1994). Rabinowitz’ framework is used by Jeliazkov et al. (2022) to build a roadmap of sampling designs, field methods and modelling approaches tailored to the distribution of rare species.

One category of rare species corresponds to being geographically widespread but occurring at low abundance within its range. For such species Jeliazkov et al. (2022) contrast a design‐based sampling design – the simplest one being random sampling in the geographical space – to convenience sampling, such as done when sampling is done close to roads or in habitats believed to be important for these rare species. The former approach will lead to unbiased estimation of species distributions and their drivers, but with many rare species would require very large resources to be precise enough to be useful for, e.g., modelling or making conservation plans. Jeliazkov et al. (2022) mention as an example the UK plants countryside survey – a survey based on stratified random sampling – which missed a large proportion of rare species. Convenience sampling may lead to more precise models of distributions but such models are likely to be biased and miss important aspects of the distribution, for example, if rare species are also found in microhabitats not sampled. It may be possible to combine approaches, by modelling the choice of sampling units and by stratifying with respect to where the species is expected to be found – for example, by using niche‐based sampling. Such model‐based designs are still rarely used in practice (Albert et al., 2010), and as Jeliaskov et al. (2022) make clear, require a good understanding of the processes leading both to where and how sampling is done.

Low abundance may also lead to concerns about detectability of species – a few individuals are harder to detect than many. Detectability will determine if a recorded absence is a true negative or a miss. But as Jeliazkov et al. (2022) carefully discuss, one difficulty is that sources of variation in detectability are likely to vary among rare species: in the same way that rare species can be rare in many different ways, low detectability may have different origins, from abundance to behavior to phenology. Standardization of field methods may reduce such variation but, in the end, one will have to carefully balance accounting for variation in detectability – i.e. complex models – and using simple and what can appear as more robust models (Yoccoz et al., 2001). Low number of individuals will also make estimation of abundance inaccurate because small sample size leads both to low precision and to the inability of assessing the assumptions of models. A solution might then be to avoid species idiosyncrasies and to develop models where similar species will share parameters. Statistical ecologists have developed a wide range of models that can share information among rare species – for example, by assuming some common shape of detectability or recapture functions – but ultimately our confidence in such models will depend on a careful integration of sampling design and field methods (Jeliazkov et al., 2022). With small number of observations per species, usual statistical criteria will favor simple models – for example, similar detectability curves – but this may reflect a lack of information rather than a real absence of important differences.

Species distribution models have become an important component of the ecological toolkit. Rare species will usually be represented by only a few observations, for example, because their geographical range is small or because their abundance is low and they are rarely detected. As for modelling of detectability or abundance estimation, small sample size may lead to simple models and large uncertainty. Jeliazkov et al. (2022) summarize some of the recent modelling developments – a daunting task given the large number of available modelling frameworks and the lack of systematic comparisons of models with respect to different objectives (e.g., understanding the current drivers of current distributions vs. predicting changes in future distributions as a consequence of climate change or changes in land cover; Briscoe et al., 2021). They rightly focus on hierarchical models that integrate modelling detectability and modelling ecological processes determining presence or abundance of a species. This may be particularly important if detectability is mainly affected by abundance – a temporal change in detectability may represent an important signal of a declining overall abundance, not some kind of measurement error that just needs to be corrected for. They therefore provide a useful entry to a large and rapidly expanding literature that will complement existing reviews.

The large number of rare species make them the dominant component of biological diversity and therefore they should play a central role in the conservation of biodiversity. Rarity is one criterion used in the classification of threatened species (Mace et al., 2008), and some categories of rarity such as low abundance and small geographical range may represent a “double jeopardy” in terms of extinction risk. However, it is not necessarily the case that management plans should focus on rare species: focusing on common species may be more cost‐effective (Neeson et al., 2018). A better understanding of the distribution of rare species may therefore help assessing the effectiveness of management plans, ideally considering diverse taxonomic groups as hotspots of rare species for one group may not coincide with hotspots for another group. Jeliazkov et al. (2022) list various field methods that can provide complementary approaches to sampling diverse taxonomic groups, from eDNA to different kinds of traps. Hierarchical models with different submodels adapted to each group and field methods may then provide an integrated view of rarity less dependent on taxonomy, and therefore a more complete approach to conservation.

Studying the importance of rare species for the functioning of ecosystems has also a long tradition in ecological research and management. We all have read about keystone species – species which are comparatively rare but have disproportionate impact on ecosystems relative to their abundance. But apart from such iconic, but relatively rare, examples, one may ask if rare species may support a diverse array of functions, and there is evidence that it might be the case (Mouillot et al., 2013). Rare species may also differ from abundant species in having positive rather than negative associations, either because of ecological interactions such as facilitation, or because of microhabitat sharing (Catalayud et al., 2020). That is, there might be some fundamental differences between rare and common species in terms of at least spatial associations and perhaps ecological interactions. Clearly, having better designs and models will help us understand the role played by rare species, both in terms of structure and functions.

Fisher is often quoted for having said that “To consult the statistician after an experiment is finished is often merely to ask him to conduct a post mortem examination. He can perhaps say what the experiment died of.” Clearly rare species pose unique challenges at all stages – from sampling design to field methods to statistical modelling. Small sample sizes, heterogeneity of rarity patterns (Rabinowitz, 1981) and spatio‐temporal changes are challenges that can easily lead to untrustworthy conclusions. Jeliazkov et al. (2022) have given us a very timely roadmap to the different decisions that one needs to consider if we are to better understand the distribution of rare species, and ultimately how we can develop reliable predictive models that can help us sustainably managing their future.

## CONFLICT OF INTEREST

The author declares no conflicts of interest.

## Data Availability

The manuscript is a commentary and does not include any data.
